# Short-Term Isokinetic Training Versus Isotonic Training: Effects on Asymmetry in Strength of Thigh Muscles

**DOI:** 10.2478/v10078-011-0070-5

**Published:** 2011-12-25

**Authors:** Dragana Golik-Peric, Miodrag Drapsin, Borislav Obradovic, Patrik Drid

**Affiliations:** 1 Laboratory of Provincial Institute for Sport, Novi Sad, Serbia; 2 Medical Faculty, University of Novi Sad, Serbia; 3 Faculty of Sport and Physical Education, University of Novi Sad, Serbia

**Keywords:** Knee joint, muscle imbalance, isokinetic training, isotonic training

## Abstract

The aim of this study was to investigate the effects of two training protocols on the isokinetic performance of athletes. The study was conducted in 38 athletes, (age 23.3 ± 3.6 years) participating in national level leagues of different sports, whose initial concentric hamstrings-to-quadriceps (conH/Q) torque ratio was lower than 0.5. During seasonal testing, an isokinetic measurement of knee extensors and flexors was performed at 60°/s. The athletes were divided into two groups. Nineteen athletes performed the isokinetic training protocol (IT) while the second group of 19 athletes followed the isotonic training protocol (RT). Both protocols lasted 4 weeks. After completing the training protocols, both groups underwent a final isokinetic testing. The isokinetic data revealed significant increases after training in measures of peak torque in both extensor and flexor muscle groups, in both the IT and RT study groups (p < 0.05). There were significant increases (p< 0.05) in conH/Q ratio in both groups after the implemented protocols, but greater in IT group. Consequently, applied IT protocol induced changes in working muscles, thereby restoring detected asymmetry to an acceptable balance more efficiently compared to RT protocol.

## Introduction

In isokinetic testing the term of asymmetry does not refer only to unequal bilateral torque values, but also to the balance between the torque ratios of agonistic and antagonistic ipsilateral muscle groups. Research papers have shown that ipsilateral ratios are valid tools to assess the effectiveness of rehabilitation protocols (Brown et al., 2000; Dvir et al., 2004; Impellizzeri et al., 2007; Drid et al., 2009a). Many papers report that bilateral differences are an important predictor of injury occurrence (Dauty et al., 2003; Yeung et al., 2009; Fousekis et al., 2010). The concentric hamstrings-to-quadriceps (conH/Q) torque ratio has been studied extensively (Perrin et al., 1993; Olmo et al., 2006) with reported averages ranging from 0.5 to 0.75.

Investigation of factors associated with muscle injuries reveal the multifactorial origin of these injuries. Among numerous causes reported in the literature, only a few have been scientifically associated with injury occurrence, while others were empirically suggested (Askling et al., 2003). Muscle strains, especially in the hamstrings, are common in athletic activities involving high acceleration. Poor flexibility is also assumed to be associated with a higher risk of hamstrings muscle injury (Brockett et al., 2004). In his recent study, Schlumberger et al. (2006) found that muscle imbalance could increase injury rate in top-level athletes. However, it appears difficult to define imbalance as a deviation from an assumed normative value because the margins of acceptable deviation are unclear (Schlumberger et al., 2006). Impellizzeri et al. (2008) pointed out that commonly used strength imbalance indices, such as the conH/Q ratio, are proper tools for monitoring rehabilitation and training interventions in non-healthy participants.

Since the introduction of isokinetics into modern rehabilitation protocols, researchers and clinicians have faced many difficulties concerning the effects and outcome of such protocols. The issues of protocol structure, such as dose, length or load, are still not settled. Despite these uncertainties, it is clear that isokinetic conditioning programs have great advantage over other conditioning and rehabilitation methods (Grimby et al., 1980; Gur et al., 2002; Tagesson et al., 2008). In contrast to weight training, maximal torque can be achieved through the whole range of motion when isokinetic training principles are applied; which may explain their larger training effect. The study of the effects of 4 weeks of isokinetic training, in a group of healthy sedentary males, found that protocol produced changes in limb acceleration, but little or no change in strength (Murray et al., 2007). This paper challenged the authors of this study to introduce the 4 week training model in individuals with registered asymmetry in strength and observe the outcome since other authors reported an increase in strength as well.

It is generally accepted that neural factors play an important role in muscle strength gains. Abe et al. (2000) demonstrated an increase in strength of upper extremity after 2 weeks of strength training while the increase in muscle mass occurred only after 4 to 6 weeks. An increase in muscular strength without noticeable hypertrophy is the first line of evidence for neural involvement in acquisition of muscular strength. This has been interpreted as an increase in neural drive, which reflects the size of neural output from the CNS to active muscle fibres (Lazaridis et al., 2010). Agonist muscles move the limb while antagonists produce countermovement. Balance between theese two groups enables smooth and well coordinated activity. The CNS will optimise both groups in order to produce as much force as posible and to maintain the integrity of the joint (Gabriel et al., 2006).

The purpose of this study was to evaluate the effect of two different training protocols, isokinetic training (IT) and isotonic training (RT), on registered initial thigh muscles strength asymmetry after 4 weeks of training.

## Methods

### Subjects

Thirty-eight male athletes participated in our study (mean age 23.3 ± 3.6 years) who were found to have strength asymmetry of thigh muscles during regular season testing among 196 atheletes examined. The inclusion criterion was a conH/Q ratio lower than 0.5. Participants represented individual sports such as judo (13.2%), wrestling (15.8%), gymnastic (7.9%), track and field (18.4%), as well as team sports such as handball (26.3%), soccer (5.3%), basketball (2.6%), and volleyball (10.5%). Two groups were formed randomly and to each group 19 participants were assigned. The first group (19 participants, 79.47 ± 16.92 kg, 182.68 ± 12.61 cm) performed 4 weeks of the IT protocol on the isokinetic dynamometer (IT group.) The second group (19 participants, 80.31 ± 10.16 kg, 183.16 ± 5.40 cm) performed 4 weeks of a RT protocol designed for the lower limbs (RT group). Medical examination of all participants, prior to the beginning of the training protocol, detected no clinical disorders of the lower limbs. All subjects gave their written consent to participate in this study and the ethics committee of Medical Faculty of Novi Sad, Serbia has approved the research.

### Testing protocol

Bilateral isokinetic dynamometry was performed (Easy Tech prima DOC; Borgo San Lorenzo (FI) ITALY) at the Department of Sport of Province of Vojvodina, Novi Sad, Serbia. This device has shown to be reliable in the collection of isokinetic data. Participants were secured to the dynamometer chair in a seated position using chest, waist, and thigh straps. The axis of rotation of the dynamometer was visually aligned with the axis of rotation of the participant's knee joint. Range of motion during testing and training was between 10° and 90° (degrees) of physiological knee flexion; 0° is full extension. The ankle pads were placed just above the subject’s lateral malleoli. Participants were instructed to keep their hands crossed in front of their chest during all testing and training sessions. Testing was conducted for both quadriceps and hamstring groups for both legs. Appropriate calibration and gravity correction were performed prior to each testing and training session. Testing was conducted at the beginning and at the end of the training protocol (lasting for 4 weeks) for both IT and RT group at 60°/s with visual and auditive feedback.

### Training protocols

The IT group participated in an individually dosed, isokinetic training protocol (IT) lasting for 4 weeks (5 sessions/wk) for both extensors and flexors of the knee (Table 1). At the beginning of each session, a warm up was performed on the dynamometer at the speed of 240°–300°/s for 3 minutes. The specific number of maximal repetitions was executed, first with the uninvolved leg, then with the one with observed asymmetry in strength. Rest periods between the sets equaled 1 minute.

The RT group participated in an individually dosed, supervised program lasting for 4 weeks (Table 1). The isotonic exercises performed by the athletes after a 10 min warm up were half-squats.

### Statistical analysis

Data were analyzed using SPSS PC (version 15.0) program for Windows. The analysis of covariance was used to test the differences between pre- and post- training data. Paired-samples T-test was used to compare within groups results. Statistical significance was set at p < 0.05.

## Results

During the regular season testing muscle imbalance or a deficit in strength was found in 38 athletes among 196 examined subjects. Among these 38, bilateral differences between the strength of muscles were found in 5% for knee extensors and in 8% for the knee flexors for the tested angular velocity of 60°/s. These findings did not indicate significant imbalance in bilateral strength. These findings did not indicate significant imbalance in strength, however, the conH/Q ratio was 0.4 or less in all athletes.

Parameters of strength of thigh muscles showed no significant variation of distribution of data. Both groups showed high homogeneity of distribution of measured variables.

Peak muscle torques of the thigh muscles of both groups, measured initially and at the end of 4 weeks, at 60°/s are presented in Table 2. There was significant improvement in strength after 4 weeks of training for all measured muscle groups (p < 0.05). There was a difference, which was statistically significant (p < 0.05), between IT and RT group for initial values of both KE-R and KEL. The initial values of isokinetic strength were higher in RT group. At the end of the 4 week training program, the differences still existed, but were no longer statistically significant.

After the assessment of thigh muscles isokinetic strength, the peak moment conH/Q ratios (%) of the right and left leg were calculated. Initial values of conH/Q ratio for both the IT and RT group were low. After the 4 weeks of implemented training protocols the values of the concentric ratios increased significantly (p < 0.05), especially for the IT group (Table 3). Furthermore, the induced changes from isokinetic training were greater than the changes observed after isotonic training (p < 0.05).

## Discussion

In recent years numerous papers, meetings and publications have addressed the topic of sport injury, its rehabilitation and possible prevention. This interest stems from the needs of everyday living as well as professional sports. In clinical and scientific research, the knee joint and thigh muscles have been amply described using a variety of techniques (Lord et al., 1992; Madsen et al., 1996; Croiser et al., 2004). In the last 30 years this region was emphasized using isokinetic testing, a useful tool to identify both physiological characteristics and pathological conditions. However, there is still no consensus regarding the importance of isokinetic predictors for injury prevention.

Low values of concentric (conH/Q) ratio have been related to a higher injury risk. Values lower than 0.47 indicate a higher injury risk (Croiser et al., 2002). In their recent paper, Houweling-Taco et al. (2009) suggested that the concentric/concentric testing method may be suitable for detecting previous hamstring injury, especially at lower speeds of testing (60°/s). Similar to previous reports, this study revealed the concentric ratio to be as low as 0.4 in all participants when tested initially (Drid et al., 2009b). Two specific training protocols were introduced in order to restore desired values of the concentric ratio and muscle strength and to evaluate the effects they induced in the trained muscle groups. The two protocols were isokinetic training (IT group) and isotonic training (RT group). After the protocols were implemented, the initial strength imbalances improved significantly in both groups. However, in the IT group these imbalances improved more than in the RT group.

The results of the research conducted by Blazevich et al. (2007) indicate that heavy isotonic exercises should be included in rehabilitation programs to induce levels of neuromuscular activation sufficient to stimulate muscle growth and strength. The mechanisms governing the increases in force production in response to short periods of strength training have yet to be fully understood. The influence of intra-session rest intervals may have a profound effect on strength gains subsequent to short-term high intensity isokinetic training (Blazevich et al., 2007). Isotonic training has been well described in the literature, and it is clear from recent reviews that the greatest gains in force and power production are seen at movement velocities similar to that of training (Caiozzo et al., 1981; Bell et al., 1992). The results of the RT group proved that this type of training can induce changes in working muscles which are statistically significant (p < 0.05), 8% and 7% for extensors (for right and left leg respectively) and 19% and 11% for flexors (for right and left leg respectively). It is interesting to notice that gains in hamstrings strength were almost twice that of the quadriceps. One possible explanation could be that initially the hamstrings were relatively weak, which is supported by the fact that the concentric ratios were initially low.

In the study of Akima et al. (1999), the effects of 2 weeks of isokinetic training are presented. The authors stated that the intensity and the load were sufficient to provoke significant changes in muscle torque production without evident hypertrophy. This was explained by increased muscle contractile activity. A similar study reported the effects of 4 weeks of isokinetic training on muscle strength and explosiveness (Murray et al., 2007). These findings disagree with the significant improvement of muscle strength obtained by IT protocol introduced in this study, for extensors 9% and 14% (for right and left leg respectively) and for flexors 30% and 27% (for right and left leg respectively). The main difference in these 4 week training protocols is that they used a total of 8 training sessions compared with 20 in this study. Therefore, the difference in training frequency could explain the gains in strength.

Assessment of lower limb strength ratios is important in detecting muscle imbalances. However, this assessment does not provide information on imbalance changes after repeated muscular contractions. In a recent study of Bampouras (2010), the author suggested that the results indicate that high intensity activity increases lower limb strength imbalance. The implication of this research, according to authors, is that assessment activities conducted during the rested state may not accurately reflect the true strength difference between limbs, leading to inaccurate training or rehabilitation protocols.

The results of this paper showed a significant increase (p<0.05) in conH/Q ratios after the implemented training protocols, but the measured changes were higher in IT group compared to RT group.

Ipsilaeral muscle asymetry and muscle imbalances close to the knee are known as the aetiology of many injuries, especially hamstrings. The questions, whether the correction of any muscle imbalance could reduce the risk of injury, or if muscle imbalance causes injury, have not been fully investigated. The peak moment conH/Q has been used to assess thigh muscle imbalance, but of its value as the predictor of the injury is open to question. Assessing muscle balance is considered important, but the peak moment ratios, if considered alone, provide limited information. Some suggest that a more functional approach to assess muscle balance is required (Coombs et al., 2002).

## Conclusion

The results of this study showed that the implemented training protocols significantly (p < 0.05) improved the strength of thigh muscles measured isokinetically and decreased the degree of muscle strength asymmetry. It is clear that the IT protocol evoked greater changes in thigh muscle strength compared with RT protocol, which is reflected in greater changes in the ipsilateral concentric ratio.

## Figures and Tables

**Table 1:** Training protocols

IT Protocol					RT Protocol			

	Set (No.)	Speed (°/s)	Repetition (No.)	Rest (min)	Set (No.)	Load (%1RM)	Repetition (No.)	Rest (min)
Week 1	6–8	300–240	15–20	2	6	60–70	15–20	2
Week 2	6–8	300–180	10–15	2	4–5	70–80	10–12	2
Week 3	6–8	240–60	10–12	1	3–4	80–90	4–6	1
Week 4	6	180 – 60	10	1	3–4	80–90	4–6	1

1 RM – 1 repetition maximum

**Table 2:** Peak muscle torques (Mean ± SD Nm) of the thigh muscles of both groups measured initially and at the end of 4 weeks at 60°/s

	IT Group		RT Group	

	Initial	Final	Initial	Final
KE-R	240.9±73.2	264.5±50.7*	264.4±40.8^e^	286.5±41.3*
KE-L	229.4±63.8	266.0±44.7*	270.4±39.8^e^	290.4±44.6*
KF-R	95.8±28.3	137.5±27.1*	101.1±21.6	124.2±29.1*
KF-L	100.0±33.7	136.6±29.7*	114.1±29.5	128.8±41.9*

Abbreviations: KE, Knee extensors; KF, Knee flexors; R, Right; L, Left;

(^e^) Significantly (p < 0.05) different from IT group.

* Significantly (p < 0.05) different from initial testing.

**Table 3:** The peak moment conH/Q ratios (%, mean ± SD) of the right and left leg

	IT Group		RT Group	

	Initial	Final	Initial	Final
R	41.8±13.7	52.4±7.9*^e^	38.6±7.0	43.6±9.3*
L	44.7±14.5	51.5±7.8*^e^	42.1±8.0	44.0±10.0*

Abbreviations: R, Right leg; L, Left leg.

* Significantly (p < 0.05) different from initial testing.

^e^ Significantly (p < 0.05) different from RT group.
